# Sex differences in LMR ratio and coronary artery stenosis severity among patients with coronary artery disease

**DOI:** 10.3389/fcvm.2026.1747052

**Published:** 2026-03-24

**Authors:** Cheng Cao, Wenyi Pang, Lingling Lu, Yang Zhao, Lu Lu, Hongmin Zhao

**Affiliations:** 1 Cardiology Department, Cangzhou Hospital of Integrated Traditional Chinese Medicine and Western Medicine of Hebei, Cangzhou, Hebei, China; 2 Oncology Department, Cangzhou Hospital of Integrated Traditional Chinese Medicine and Western Medicine of Hebei, Cangzhou, Hebei, China

**Keywords:** coronary artery disease, coronary artery stenosis severity, gensini score, lymphocyte-to-monocyte ratio, sex differences

## Abstract

**Background:**

Previous studies have indicated that the lymphocyte-to-monocyte ratio (LMR) is associated with the severity of coronary artery disease (CAD). However, whether this association differs between sexes remains unclear. This study aimed to evaluate the association between LMR and Gensini score in patients with CAD, with a focus on potential sex differences.

**Methods:**

A total of 1,673 patients undergoing coronary angiography were included. The severity of CAD was assessed using the Gensini score, and patients were categorized into three groups based on Gensini score tertiles: T1 (≤10), T2 (>10 to <44), and T3 (≥44). Sex-stratified analyses and multivariable regression models were employed to assess the association between LMR and CAD severity.

**Results:**

LMR was significantly lower in patients with higher Gensini scores (*P* < 0.001). After multivariable adjustment, LMR was independently and inversely associated with the Gensini score in the overall population. A significant sex–LMR interaction was observed (*P* < 0.05). In sex-stratified analyses, the inverse association between LMR and Gensini score was stronger in females than in males.

**Conclusion:**

LMR is independently and inversely associated with the severity of CAD, with a stronger association observed in female patients. These findings suggest that LMR may serve as a potential inflammatory marker for assessing CAD severity, and its clinical relevance may differ between sexes.

## 1 Introduction

Coronary Artery Disease (CAD) is a chronic condition caused by atherosclerotic plaques that narrow the coronary arteries. It is characterized by persistent inflammation in the vessel walls, which contributes to the progression of plaque buildup and further narrowing, ultimately leading to myocardial ischemia, hypoxia, and potential tissue necrosis (1). Despite significant advances in diagnostic and therapeutic technologies, the incidence and burden of CAD remain high (2). Research suggests that coronary artery stenosis is not merely a mechanical obstruction but the result of the dynamic progression of atherosclerotic plaques driven by inflammation (3). Therefore, exploring biomarkers that accurately reflect the severity of coronary artery narrowing and the activity of inflammation is crucial for risk stratification and personalized intervention.

Coronary Angiography (CAG) is considered the gold standard for diagnosing CAD clinically, providing a quantifiable assessment of stenosis severity and lesion extent (3). The Gensini score system, based on CAG, offers a comprehensive evaluation of coronary artery lesion severity by integrating lesion location, stenosis degree, and calcification characteristics (4). It is a key tool for determining revascularization strategies and predicting prognosis. However, as an invasive procedure, the application of CAG is limited and difficult to generalize, especially in high-risk populations with multiple risk factors (such as diabetic patients) for early screening.

Inflammation plays a key role throughout the development of CAD (5). Both lymphocyte-mediated adaptive immunity and monocyte-driven innate immunity imbalance play pivotal roles in the inflammatory response of atherosclerosis (6). A decrease in lymphocytes often indicates a weakened anti-inflammatory and immune surveillance function, whereas an increase in monocytes is closely associated with the release of pro-inflammatory mediators, plaque instability, and lesion progression (7). The Lymphocyte-to-Monocyte Ratio (LMR), a composite inflammatory marker, can dynamically reflect the relative balance between these two key immune cell types. Existing studies have confirmed that low LMR is significantly associated with the risk of adverse cardiovascular events such as acute coronary syndrome and heart failure (8). However, these studies have primarily focused on the prognostic value of LMR in the long term, and systematic research on its direct correlation with the severity of coronary artery stenosis, particularly considering potential sex differences, remains lacking.

Sex is an important factor influencing the occurrence and progression of CAD (9). Epidemiological studies show that the incidence of CAD is higher in men than in women, but post-menopausal women experience faster disease progression and worse prognosis. This difference may be related to the regulatory effects of sex hormones on the inflammatory response (10). Estrogen reduces the risk of CAD by improving endothelial function and lipid metabolism, while enhancing T-cell-mediated immune responses, increasing lymphocyte count, and inhibiting monocyte-driven pro-inflammatory responses. In contrast, inflammation in male patients is more likely to involve an increase in monocytes and elevated levels of pro-inflammatory cytokines. Therefore, sex may play a crucial modulating role in the relationship between LMR and the degree of coronary artery stenosis, but this hypothesis has not been conclusively verified.

Although LMR has been shown to be associated with the prognosis of CAD patients, most studies focus on the risk of cardiovascular events without delving into its relationship with the severity of coronary artery stenosis. Moreover, existing research has not conducted sex-stratified analyses, overlooking the potential interaction between sex, LMR, and CAD. Therefore, this study aims to systematically explore, for the first time, the relationship between LMR and the severity of coronary artery stenosis quantified by the Gensini score in a large cohort of CAD patients. It will also investigate the potential role of sex in this relationship, hoping to provide evidence for personalized risk assessment in CAD patients and offer new perspectives on the immunological mechanisms underlying sex differences in CAD.

## 2 Methods

### 2.1 Patients and study design

This was a retrospective, single-centre, cross-sectional observational study. We screened consecutive patients who were admitted to the Department of Cardiology Cang zhou Hospital of Integrated Traditional Chinese and Western of Hebei Province between March 2021 and January 2025. All patients presented with typical chest pain or chest discomfort and subsequently underwent diagnostic CAG. A total of 1 673 individuals (911 men and 762 women) fulfilled the inclusion criteria. Exclusion criteria were: (1) incomplete medical records; (2) persistent or paroxysmal atrial fibrillation; (3) severe hepatic or renal insufficiency, significant valvular heart disease, cardiomyopathy, rheumatic heart disease, or malignancy with concurrent infection. The study protocol was approved by the hospital's Ethics Committee (approval No. CZX2025-KY087) and was conducted in accordance with the Declaration of Helsinki. Because the study was retrospective and involved no additional interventions, patient data were anonymised to ensure confidentiality; the requirement for informed consent was therefore waived.

### 2.2 Data collection and measurements

Demographic and clinical data—including sex, age, body-mass index (BMI), systolic and diastolic blood pressure, history of hypertension, history of diabetes mellitus, and smoking status—were extracted from the hospital's electronic medical-record system. After an overnight fast, venous blood samples were collected on the morning following admission for routine laboratory analyses. Measured variables comprised lymphocyte count (LYMP), monocyte count (MONO), alanine aminotransferase (ALT), aspartate aminotransferase (AST), total cholesterol (TC), triglycerides (TG), high-density-lipoprotein cholesterol (HDL-C), low-density-lipoprotein cholesterol (LDL-C), and fasting blood glucose (FBG). The lymphocyte-to-monocyte ratio (LMR) was calculated as LYMP (×10^9^/L) divided by MONO (×10^9^/L).

### 2.3 Coronary angiography

Before coronary angiography or percutaneous coronary intervention (PCI), all participants were treated with a loading dose of 300 mg aspirin and 300 mg clopidogrel. All procedures were performed by board-certified interventional cardiologists. In this study, vascular access was consistently obtained via the radial artery. The imaging protocol followed the Judkins technique, involving multiple angiographic projections of both the left and right coronary systems to ensure thorough visualization of the most significant stenoses.

To ensure the objectivity and accuracy of the results, the anatomical location and quantitative severity of coronary lesions were independently assessed and recorded by two or three experienced clinicians. These clinicians were explicitly blinded to all laboratory data, including LMR values, during the angiographic evaluation. The diagnosis of CAD was made according to the current guidelines of the American College of Cardiology (ACC) and the American Heart Association (AHA): a diameter stenosis ≥30% in the left main coronary artery, or ≥50% in any other major epicardial branch, was considered diagnostic of CA (1). The extent of atherosclerotic burden was further categorized using the Gensini scoring system.

### 2.4 Definition

The anatomical severity of CAD was evaluated based on both the number of epicardial vessels involved and the Gensini score. The Gensini score is a validated angiographic metric that quantifies the degree of luminal narrowing and incorporates the functional significance of lesion location (11). Lesions were assigned points based on the percentage of diameter stenosis: <25% stenosis received 1 point, 26%–50% received 2 points, 51%–75% received 4 points, 76%–90% received 8 points, 91%–99% received 16 points, and total occlusion (100%) received 32 points. These segmental scores were then adjusted by a weighting factor reflecting the myocardial mass at risk in each area: 5 for the left main coronary artery, 2.5 for the proximal left anterior descending or circumflex artery, and 1.5 for the mid-portion of the left anterior descending artery.

Finally, the Gensini score was calculated by summing the scores of individual coronary segments (12, 13). For analytical purposes, patients were stratified into three groups according to tertiles of the Gensini score: T1 (≤10), T2 (>10 to <44), and T3 (≥44).

### 2.5 Statistical analysis

All analyses were performed with SPSS 27.0 and Stata 17.0, and two-tailed *P* < 0.05 was considered statistically significant. Normality of continuous variables was first examined with the Shapiro–Wilk test. Normally distributed data are presented as mean ± SD, whereas non-normally distributed data are expressed as median (inter-quartile range, IQR). Categorical variables are summarised as frequencies and percentages (%). For baseline comparisons, normally distributed continuous variables were compared between two groups with the independent-samples t test or across multiple groups with one-way ANOVA; otherwise, the Mann–Whitney U or Kruskal–Wallis test was employed. Categorical variables were evaluated with the *χ*² test or Fisher's exact test, as appropriate. Correlation strength was quantified with Pearson's correlation coefficient (r) when the data met normality and linearity assumptions. Otherwise, Spearman's rank correlation coefficient (*ρ*) was used. The *P* value represents the probability associated with the corresponding correlation test.

A multiple linear regression model was utilized to examine the relationship between LMR and Gensini scores. To delve into the differences in this association across sex groups, the SUEST test was employed for statistical analysis. This test enables hypothesis testing among regression models with potentially correlated error structures. By comparing the regression coefficients between male and female cohorts, we can systematically determine whether the impact of LMR on Gensini scores shows sex specificity (14).

## 3 Results

### 3.1 Baseline characteristics

This study included a total of 1,673 patients with coronary heart disease, comprising 911 males and 762 females. The average age of the study population was 64.01 ± 7.92 years, with an average BMI of 25.66 ± 3.36 kg/m^2^, an average SBP of 136.57 ± 17.32 mmHg, and an average DBP of 78.59 ± 11.70 mmHg. Regarding vascular risk factors, 45.0% and 33.4% of participants were current smokers and drinkers, respectively. Additionally, 62.0% had hypertension, and 29.2% had diabetes mellitus. A family history of coronary heart disease was present in 23.3% of the participants, and a history of stroke in 14.0%. In terms of disease history, the prevalence of antidiabetic medication use at enrollment was 27.6%, antihypertensive medication use was 61.6%, and lipid-lowering medication use was 11.9%.

The median Gensini score was 22.00 (IQR: 10.00–44.0), and the median LYMP was 3.32 (IQR: 3.25–3.41). Sex difference analysis showed that age, DBP, smoking, drinking, use of antihypertensive drugs, Gensini score, LYMP, MONO, LMR, ALT, AST, TG, TC, HDL, and LDL were statistically significant (*P* < 0.05). Details are provided in Table 1.

**Table 1:** Basic characteristics of the research object.

Variable	Total (*n* = 1,673)	Male (*n* = 911)	Female (*n* = 762)	statistical value	*P* value
Age, year (SD)	64.01 ± 7.92	62.34 ± 8.46	66.04 ± 6.69	*t* = −9.781	<0.001*
BMI, kg/m^2^ (SD)	25.66 ± 3.36	25.73 ± 3.27	25.59 ± 3.46	*t* = 0.864	0.388
SBP, mmHg (SD)	136.57 ± 17.32	136.68 ± 17.21	136.43 ± 17.46	*t* = 0.303	0.762
DBP, mmHg (SD)	78.59 ± 11.70	79.68 ± 11.71	77.27 ± 11.55	*t* = 4.212	<0.001*
Smoking, *n* (%)	753 (45.0%)	643 (70.6%)	110 (14.4%)	*χ*^2^ = 528.476	<0.001*
Drinking, *n* (%)	558 (33.4%)	505 (55.4%)	53 (7.0%)	χ^2^ = 438.626	<0.001*
Hypertension, *n* (%)	1,037 (62.0%)	545 (59.8%)	492 (64.6%)	χ^2^ = 3.961	0.047
Diabetes, *n* (%)	488 (29.2%)	260 (28.5%)	228 (29.9%)	χ^2^ = 0.383	0.536
Family history of CAD, *n* (%)	390 (23.3%)	209 (22.9%)	181 (23.8%)	χ^2^ = 0,153	0.696
History of stroke, *n* (%)	234 (14.0%)	140 (15.4%)	94 (12.3%)	χ^2^ = 3.170	0.075
Medication history					
Antidiabetic drugs, *n* (%)	461 (27.6%)	242 (26.6%)	219 (28.7%)	χ^2^ = 0.984	0.321
Antihypertensive drugs, *n* (%)	1,030 (61.6%)	531 (58.3%)	499 (65.5%)	χ^2^ = 9.085	0.003*
Lipid-lowering drugs, *n* (%)	199 (11.9%)	110 (12.1%)	89 (11.7%)	χ^2^ = 0.062	0.804
Gensini score (IQR)	22.00 [10.00–44.0]	27.00 [12.00–50.00]	16.00 [8.00- 3.00]	*Z* = −8.945	<0.001*
LYMP, 10^9^/L(IQR)	1.81 [1.41–2.37]	1.79 [1.36–2.34]	1.83 [1.45–2.42]	*Z* = 2.025	0.043*
MONO, 10^9^/L (IQR)	0.45 [0.34–0.62]	0.48 [0.38–0.68]	0.40 [0.31–0.54]	*Z* = −9.086	<0.001*
LMR, 10^9^/L (IQR)	3.32 [3.25–3.41]	3.32 [3.25–3.40]	3.33 [3.24–3.43]	*Z* = 6.010	<0.001*
FBG, mmol/L (IQR)	5.83 [5.28–6.99]	5.81 [5.26–6.93]	5.87 [5.28–7.07]	*Z* = 0.793	0.428
ALT, U/L (IQR)	18.00 [13.00–26.95]	19.70 [14.45–29.00]	17.00 [12.00–24.00]	*Z* = −6.149	<0.001*
AST, U/L (IQR)	19.00 [15.00–24.00]	19.00 [15.00–26.00]	18.95 [15.00–23.00]	*Z* = −1.855	0.064
TG, mmol/L (IQR)	1.45 [1.06–2.08]	1.45 [1.06–2.12]	1.47 [1.06–2.05]	*Z* = 8.440	<0.001*
TC, mmol/L (SD)	4.58 ± 1.11	4.37 ± 1.03	4.83 ± 1.16	*t* = −8.607	<0.001*
HDL, mmol/L (IQR)	1.11 [0.96–1.30]	1.04 [0.89–1.19]	1.19 [1.05–1.38]	*Z* = 13.175	<0.001*
LDL, mmol/L (SD)	2.91 ± 0.91	2.79 ± 0.86	3.05 ± 0.95	*t* = −5.867	<0.001*

SBP, systolic blood pressure; DBP, diastolic blood pressure, FBG, fasting blood glucose, LYMP, lymphocytes, MONO, monocytes, LMR, lymphocyte to monocyte ratio, ALT, alanine aminotransferase, AST, aspartate aminotransferase, TG, Triglycerides, TC, total cholesterol, HDL, high-density lipoprotein, LDL, low-density lipoprotein.

* *P* < 0.05 (two-sided) was considered statistically significant.

In addition, participants were divided into three groups based on the tertiles of their Gensini scores: T1 (≤10), T2 (10–44), and T3 (≥44). Differences in general demographic data among the three groups were statistically significant (*P* < 0.05) in terms of participants' sex, systolic blood pressure, smoking and drinking history, hypertension, diabetes, stroke history, and prior use of antihypertensive and antidiabetic medications. Regarding clinical biochemical indicators, statistically significant differences (*P* < 0.05) were found across the groups for LYMP, MONO, LMR, FBG, ALT, AST, TG, and HDL (Table 2).

**Table 2:** Characteristics of individuals categorized by tertiles of Gensini scores.

Variable	Gensini			Statistics	*P* value
	T1 (Gensini ≤ 10, *n* = 462)	T2 (10 < Gensini < 44, *n* = 806)	T3 (Gensini ≥ 44, *n* = 434)		
Age, year (SD)	64.38 ± 6.97	64.06 ± 8.16	63.54 ± 8.41	*F* = 1.250	0.287
Sex, *n* (%)				χ^2^ = 67.415	<0.001*
Male	196 (43.1%)	447 (56.3%)	268 (63.2%)	χ^2^ = 37.933	<0.001*
Female	259 (56.9%)	347 (43.7%)	156 (36.8%)		
BMI, kg/m^2^ (SD)	25.39 ± 3.55	25.80 ± 3.35	25.71 ± 3.14	*F* = 2.239	1.107
SBP, mmHg (SD)	133.74 ± 15.68	137.05 ± 17.58	138.69 ± 18.13	*F* = 9.656	<0.001*
DBP, mmHg (SD)	77.71 ± 11.21	78.53 ± 11.73	79.67 ± 12.07	*F* = 3.145	0.043
Smoking, *n* (%)	157 (34.5%)	376 (47.4%)	220 (51.9%)	χ^2^ = 30.150	<0.001*
Drinking, *n* (%)	109 (24.0%)	291 (36.6%)	158 (37.3%)	χ^2^ = 24.875	<0.001*
Hypertension, *n* (%)	265 (58.2%)	505 (63.6%)	283 (66.7%)	χ^2^ = 7.087	0.029*
Diabetes, *n* (%)	99 (21.8%)	236 (29.7%)	153 (36.1%)	χ^2^ = 22.028	<0.001*
Family history of CAD, *n* (%)	111 (24.4%)	174 (21.9%)	105 (24.8%)	χ^2^ = 1.667	0.435
History of stroke, *n* (%)	54 (11.9%)	103 (13.0%)	77 (18.2%)	χ^2^ = 8.516	0.014
Medication history					
Antidiabetic drugs, *n* (%)	87 (19.1%)	226 (28.5%)	148 (34.9%)	χ^2^ = 28.018	<0.001*
Antihypertensive drugs, *n* (%)	260 (57.1%)	492 (62.0%)	278 (65.6%)	χ^2^ = 6.682	0.035*
Lipid-lowering drugs, *n* (%)	59 (13.0%)	95 (12.0%)	45 (10.6%)	χ^2^ = 1.167	0.558
LYMP, 10^9^/L (IQR)	1.84 [1.44–2.50]	1.81 [1.42–2.35]	1.77 [1.35–2.28]	*Z* = 6.743	0.034*
MONO, 10^9^/L (IQR)	0.41 [0.33–0.57]	0.46 [0.33–0.62]	0.46 [0.37–0.67]	Z = 19.778	<0.001*
LMR, 10^9^/L (IQR)	3.40 [3.26–3.78]	3.33 [3.26–3.37]	3.27 [3.21–3.36]	*Z* = 20.753	<0.001*
FBG, mmol/L (IQR)	5.61 [5.16–6.54]	5.86 [5.30–6.96]	6.13 [5.34–7.75]	*Z* = 37.844	<0.001*
ALT, U/L (IQR)	17.00[12.00–24.00]	18.00 [13.00–26.00]	19.95 [15.00–30.75]	*Z* = 18.205	<0.001*
AST, U/L (IQR)	18.00 [15.00–21.80]	18.65 [15.00–24.00]	20.00 [16.00–30.00	Z = 27.031	<0.001*
TG, mmol/L (IQR)	1.34 [0.97–1.89]	1.45 [1.07–2.11]	1.60 [1.11–2.28]	*Z* = 25.079	<0.001*
TC. mmol/L (SD)	4.58 ± 1.06	4.55 ± 1.11	4.64 ± 1.17	*F* = 0.796	0.475
HDL, mmol/L (IQR)	1.18 [1.02–1.36]	1.10 [0.94–1.29]	1.06 [0.93–1.22]	*Z* = 51.689	<0.001*
LDL, mmol/L (SD)	2.89 ± 0.88	2.88 ± 0.91	2.99 ± 0.95	*F* = 02.327	0.096

SBP, systolic blood pressure; DBP, diastolic blood pressure; FBG, fasting blood glucose; LYMP, lymphocytes; MONO, monocytes; LMR, lymphocyte to monocyte ratio; ALT, alanine aminotransferase; AST, aspartate aminotransferase; TG, triglycerides; TC, total cholesterol; HDL, high-density lipoprotein; LDL, low-density lipoprotein.

* *P* < 0.05 (two-sided) was considered statistically significant.

### 3.2 Correlation of clinical characteristics with LMR ratio and gensini score

Using Spearman correlation analysis, the correlation coefficients of Clinical Characteristics with LMR Ratio and Gensini Score were calculated. The results showed significant correlations between LMR and Gensini score (*ρ* = −0.296, *P* < 0.001), SBP (*ρ* = −0.195, *P* < 0.001), DBP (*ρ* = −0.154, *P* = 0.027), LYMP (*ρ* = 0.123, *P* = 0.051), MONO (*ρ* = −0.171, *P* = 0.004), ALT (*ρ* = −0.169, *P* = 0.005), AST (*ρ* = −0.176, *P* = 0.002), TG (*ρ* = −0.190, *P* < 0.001), HDL (*ρ* = 0.210, *P* < 0.001), LDL (*ρ* = −0.156, *P* = 0.022). Gensini score also showed significant correlations with SBP (*ρ* = 0.186, *P* < 0.001), LYMP (*ρ* = −0.181, *P* < 0.001), MONO (*ρ* = 0.209, *P* < 0.001), FBG (*ρ* = 0.167, *P* < 0.001), ALT (*ρ* = 0.198, *P* < 0.001), AST (*ρ* = 0.121, *P* < 0.001), TG (*ρ* = 0.126, *P* < 0.001), HDL (*ρ* = −0.186, *P* < 0.001), as shown in Table 3.

**Table 3:** Correlation of clinical characteristics with lmr ratio and gensini score.

Variable	LMR		Gensini	
	*ρ*-value	* P* value	ρ-value	* P* value
Age, year	0.107	0.021*	−0.027	0.266
BMI, kg/m^2^	0.002	0.928	0.027	0.264
SBP, mmHg	−0.195	<0.001*	0.186	<0.001*
DBP, mmHg	−0.154	0.027*	0.051	0.056
LYMP, 10^9^/L	0.123	0.051	−0.181	0.001*
MONO, 10^9^/L	−0.171	0.004*	0.209	<0.001*
FBG (mmol/L)	−0.188	<0.001*	0.167	<0.001*
ALT (mmol/L)	−0.169	0.005	0.198	<0.001*
AST (mmol/L)	−0.176	0.002	0.121	<0.001*
TC (mmol/L)	−0.028	0.256	0.003	0.909
TG (mmol/L)	−0.190	<0.001*	0.126	<0.001*
HDL (mmol/L)	0.210	<0.001*	−0.186	<0.001*
LDL (mmol/L)	−0.156	0.022*	0.031	0.206
Gensini score	−0.296	<0.001*	-	-

SBP, systolic blood pressure; DBP, diastolic blood pressure; FBG, fasting blood glucose; LYMP, lymphocytes; MONO, monocytes; LMR, lymphocyte to monocyte ratio; ALT, alanine aminotransferase; AST, aspartate aminotransferase; TG, triglycerides; TC, total cholesterol; HDL, high-density lipoprotein; LDL, low-density lipoprotein.

*r* indicates Pearson's correlation coefficient; *ρ* indicates Spearman's rank correlation coefficient.

* *P* < 0.05 (two-sided) was considered statistically significant.

### 3.3 The relationship between LMR ratio and the degree of CAS

The results of the multivariate linear regression show that the Gensini score is independently associated with LMR, sex, history of stroke, family history of CAD, FBG, AST, and HDL (*P* < 0.05). The variance inflation factor (VIF) values for all independent variables range from 1.028 to 6.648, indicating no significant multicollinearity among the variables, as shown in Table 4.

**Table 4:** Multivariate linear regression with gensini score as the dependent variable.

Variable	B	SD	*β*	t	95%CI	*P* value	VIF
LMR, 10^9^/L	−34.643	3.611	−0.522	−9.526	−41.764 to −31.522	<0.001*	1.079
Sex	−4.573	1.639	−0.076	−2.79	−7.787 to −1.358	0.005*	1.894
Age, year	0.18	0.084	0.047	2.143	0.015–0.344	0.062	1.254
BMI, kg/m^2^	−0.19	0.187	−0.021	−1.013	−0.557–0.178	0.311	1.126
SBP, mmHg	−0.009	0.046	−0.005	−0.195	−0.099–0.081	0.845	1.802
DBP, mmHg	0.056	0.068	0.022	0.829	−0.077–0.190	0.407	1.805
Smoking	1.946	1.689	0.032	1.152	−1.367–5.258	0.249	2.013
Drinking	−2.901	1.716	−0.045	−1.69	−6.266–0.467	0.091	1.873
Hypertension	0.397	3.195	0.006	0.124	−5.869–6.663	0.901	6.648
Diabetes	2.206	3.199	0.033	0.69	−4.068–8.480	0.491	6.091
History of stroke	4.598	1.75	0.053	2.628	1.166–8.031	0.009*	1.049
Family history of CAD	2.979	1.421	0.042	2.096	0.191–5.767	0.036*	1.028
Medication history							
Antidiabetic drugs	−2.52	3.339	−0.037	−0.755	−9.069–4.029	0.45	6.337
Antihypertensive drugs	1.078	3.211	0.017	0.336	−5.221–7.377	0.737	6.448
Lipid-lowering drugs	0.643	1.873	0.007	0.343	−3.032–4.317	0.732	1.047
FBG (mmol/L)	1.407	0.356	0.089	3.952	0.709–2.105	<0.001*	1.315
ALT, U/L	−0.036	0.035	−0.023	−1.026	−0.104–0.033	0.305	1.318
AST, U/L	0.175	0.028	0.139	6.213	0.120–0.230	<0.001*	1.289
TC (mmol/L)	1.479	1.304	0.055	1.134	−1.079–4.036	0.257	5.999
TG (mmol/L)	0.565	0.484	0.026	1.169	−0.383–1.514	0.243	1.248
HDL (mmol/L)	−5.395	2.568	−0.052	−2.101	−10.431 to −0.359	0.036*	1.593
LDL (mmol/L)	−0.474	1.479	−0.014	−0.321	−3.375–.426	0.749	5.158

Multivariable model adjusted for demographic, clinical, medication, and laboratory variables listed in the table.

* *P* < 0.05 (two-sided) was considered statistically significant.

Subgroup analysis results reveal a significant sex interaction in the relationship between LMR and the degree of CAS (*P* < 0.001). After adjusting for risk factors, the impact of LMR on the Gensini score shows sex differences (*β* male = −2.14, *β* female = −8.68), and this difference is statistically significant (*P* = 0.039), as shown in Table 5.

**Table 5:** Multivariate linear regression analysis of LMR and degree of CAS stratified by sex.

Group	B	SD	β	t	95%CI	*P* value of LMR	*P* value of SUEST test
Model 0							<0.001*
Female	−30.582	4.386	−6.97	−2.84	−39.179 to −21.985	0.002*	
Male	−47.145	3.635	−12.97	−7.86	−54.271 to −40.020	<0.001*	
Model 1							0.002*
Female	−43.628	3.846	−11.34	−7.25	−51.167 to −36.089	<0.001*	
Male	−19.512	6.637	−2.94	−2.74	−32.520 to −6.505	0.003*	
Model 2							0.039*
Female	−28.710	6.135	−8.68	−4.68	−40.753 to −16.667	<0.001*	
Male	−18.226	6.791	−2.14	−2.68	−31.555 to −4.896	0.007*	

Model 0: Unadjusted; Model 1: Adjusted for sex, age, BMI, SBP, DBP, smoking, drinking, hypertension, diabetes, history of stroke, family history of coronary heart disease, and medications for lowering blood pressure, blood sugar, and lipids; Model 2: Based on Model 1, further adjusted by adding FBG, ALT, AST, TC, TG, HDL, and LDL.

* *P* < 0.05 (two-sided) was considered statistically significant.

## 4 Discussion

In this large cohort of 1,673 patients with CAD, LMR was significantly and independently associated with the severity of coronary artery stenosis as assessed by the Gensini score, and this association was observed in both men and women. After multivariable adjustment, higher LMR values were independently associated with lower Gensini scores, indicating that patients with relatively higher LMR levels exhibited substantially less severe coronary stenosis (B = −23.230, 95% CI: −32.206 to −14.255, *P* < 0.001). Sex-stratified analyses further revealed that this inverse association was more pronounced in women than in men (female *β* = −8.68; male *β* = −2.14; *P* for SUEST = 0.039), suggesting a sex-related difference in the strength of association rather than a sex-exclusive effect.

Previous studies have reported that a low LMR is associated with the onset and poor prognosis of CAD. It has been identified as an independent predictor of cardiovascular events such as acute coronary syndrome and heart failure (15). In early-onset CAD patients, an LMR < 4.18, when accompanied by elevated lipoprotein (a) [Lp(a)] levels, has been linked to the progression of carotid atherosclerosis (16). Similarly, other studies have shown that CAD patients tend to have lower LMR values compared to non-CAD patients. LMR has also been negatively correlated with indices of coronary disease severity, such as calcification scores (17). However, most of these investigations have focused primarily on clinical outcomes rather than angiographically quantified coronary stenosis severity. Moreover, formal statistical testing of sex–LMR interaction has rarely been conducted. In this context, our findings provide additional evidence supporting an association between LMR and anatomical coronary burden as assessed by the Gensini score. Unlike previous outcome-based studies, our analysis specifically evaluated angiographically quantified stenosis severity and formally tested the interaction between sex and LMR using the SUEST method. We acknowledge that several other immune cell-derived inflammatory indices, including neutrophil-to-lymphocyte ratio (NLR) and platelet-to-lymphocyte ratio (PLR), have been reported to be associated with cardiovascular risk (18). The present study was primarily designed to investigate the association between LMR and angiographic coronary burden rather than to perform head-to-head comparisons among inflammatory biomarkers. Moreover, neutrophil-related parameters were not systematically available for all participants, which precluded a robust comparative analysis. Therefore, we cannot determine whether LMR provides incremental predictive value beyond other established inflammatory indices. Importantly, LMR is derived from routinely available complete blood count parameters and does not require additional laboratory testing (19). This practical advantage may facilitate its incorporation into routine clinical assessment. Nevertheless, future prospective studies incorporating multiple inflammatory biomarkers are needed to clarify their relative and incremental predictive utilities.

LMR, as an inflammatory biomarker, reflects the balance between adaptive immunity, represented by lymphocytes, and innate immune activation mediated by monocytes. Given the central role of immune dysregulation in atherosclerosis (20), lymphopenia, particularly reduced regulatory T-cell populations, has been linked to enhanced inflammatory responses and disease progression (21). Conversely, increased monocyte counts are related to macrophage differentiation and foam cell formation within atherosclerotic plaques (22). In addition to reflecting immune functional balance, LMR may also partially mirror underlying hematopoietic dynamics. It has been reported that chronic inflammatory states, including CAD, are characterized by enhanced myelopoiesis at the expense of lymphopoiesis. Such inflammation-driven lineage shifts may lead to increased circulating monocytes and relatively reduced lymphocyte levels, thereby lowering the LMR. In this framework, lower LMR values may be indicative of a pro-inflammatory milieu associated with greater plaque burden. Nonetheless, several limitations necessitate a nuanced interpretation of these findings. Specifically, the absence of direct measurements for systemic inflammatory mediators—such as hs-CRP, IL-6, or TNF-α—and the lack of characterization regarding lymphocyte subpopulations or monocyte activation states limit our ability to draw mechanistic conclusions. Consequently, while our results align with established biological plausibility, they primarily establish an association between LMR and angiographic severity rather than confirming causal pathways. Further research is required to elucidate the underlying molecular mechanisms.

A notable finding of this study was the sex-based heterogeneity in the association between LMR and the Gensini score. Although male patients exhibited higher exposure rates to traditional risk factors such as smoking and alcohol consumption, the inverse association between LMR and coronary stenosis severity appeared stronger in women. The interaction between sex and LMR remained statistically significant after multivariable adjustment, indicating differential effect magnitude rather than exclusivity. However, an important consideration is the age difference between sexes in our cohort. Female participants were, on average, older than male participants, and most were likely postmenopausal. Since we did not collect data on menopausal status or measure serum estrogen levels, we cannot determine the extent to which hormonal factors contributed to the observed sex differences. Age-related immune senescence and alterations in lymphocyte–monocyte dynamics may also play a role. Although age was included as a covariate in multivariable models, residual confounding cannot be excluded. Existing literature suggests that estrogen is associated with modulation of lipid metabolism, endothelial function, and immune regulation (23, 24). Declining estrogen levels after menopause have been linked to changes in inflammatory profiles and metabolic parameters (25). These findings provide a plausible biological context. However, in the absence of direct hormonal measurements, these considerations remain hypothetical in the present study.

Anatomical differences between men and women may also contribute to the observed heterogeneity. Previous studies have reported smaller average coronary artery diameters in women (26). Under comparable plaque burden, smaller vessel size may be associated with relatively greater luminal narrowing. This anatomical context may partly contribute to the observed sex-specific association between LMR and stenosis severity. However, coronary vessel dimensions were not directly assessed in our analysis.

## 5 Limitations and future directions

When elaborating on the research findings, the following limitations must be acknowledged. First, the single-center, cross-sectional design limits causal inference, and the observed associations do not establish temporal relationships. Prospective cohort studies are needed to clarify the longitudinal and prognostic value of LMR in CAD. Second, serum estrogen levels, menopausal status, and key inflammatory markers (e.g., CRP, IL-6, TNF-α) were not assessed, restricting mechanistic interpretation of the observed sex-specific associations. Future studies incorporating hormonal evaluation and detailed immune profiling may help elucidate the underlying biological pathways. Third, LMR was measured only once at admission, which may reflect acute inflammatory status rather than chronic immune balance. Serial assessments could provide further insight into its dynamic clinical significance. Fourth, the study population was derived from a single ethnic and healthcare setting, potentially limiting generalizability. Multicenter studies in diverse populations are warranted to validate these findings. Finally, the absence of direct comparisons between LMR and other established inflammatory indices, such as NLR or PLR, limits conclusions regarding its incremental predictive value. Given its low cost and wide availability from routine blood testing, LMR may serve as a complementary marker within comprehensive risk assessment models. However, it should not replace imaging-based evaluation. Further prospective validation is required to define its role in risk stratification and clinical decision-making.

## 6 Conclusion

The present findings indicate that LMR was independently associated with the angiographic severity of coronary artery disease, with a distinct sex-specific pattern. These results highlight the importance of systemic immune balance in the pathophysiology of coronary atherosclerosis. The observed sex differences further suggest potential contributions of hormonal and immunological mechanisms. Future mechanistic and prospective studies are warranted to validate these associations and to determine the clinical utility of LMR in cardiovascular risk stratification.

## Data Availability

The raw data supporting the conclusions of this article will be made available by the authors, without undue reservation.
